# Dextran Sodium Sulfate-Induced Impairment of Protein Trafficking and Alterations in Membrane Composition in Intestinal Caco-2 Cell Line

**DOI:** 10.3390/ijms21082726

**Published:** 2020-04-15

**Authors:** Mohamad Toutounji, Dalanda Wanes, Mohammad El-Harakeh, Marwan El-Sabban, Sandra Rizk, Hassan Y. Naim

**Affiliations:** 1 Department of Physiological Chemistry, University of Veterinary Medicine Hannover, Bünteweg 17, 30559 Hannover, Germany; mohamadtoutounji@msn.com (M.T.); dalanda.wanes@tiho-hannover.de (D.W.); 2 Department of Anatomy, Cell Biology, and Physiological Sciences, Faculty of Medicine, American University of Beirut, Beirut 11-0236, Lebanon; mohammadelharake@gmail.com (M.E.-H.); me00@aub.edu.lb (M.E.-S.); 3 Department of Natural Sciences, Lebanese American University, Beirut 1102-2801, Lebanon; sandra.rizk@lau.edu.lb

**Keywords:** endoplasmic reticulum stress, anti- and pro-inflammatory cytokines, inflammatory bowel disease, cholesterol, brush border membranes, sucrase-isomaltase, dipeptidyl peptidase-4, lipid rafts

## Abstract

A key morphological feature of inflammatory bowel disease (IBD) is the loss of the barrier function of intestinal epithelial cells. The present study investigates endoplasmic reticulum (ER) stress in addition to alterations in protein and membrane trafficking in a dextran sulfate sodium (DSS)-induced IBD-like phenotype of intestinal Caco-2 cells in culture. DSS treatment significantly reduced the transepithelial electric resistance (TEER) and increased the epithelial permeability of Caco-2 cells, without affecting their viability. This was associated with an alteration in the expression levels of inflammatory factors in addition to an increase in the expression of the ER stress protein markers, namely immunoglobulin-binding protein (BiP), C/EBP homologous protein (CHOP), activation transcription factor 4 (ATF4), and X-box binding protein (XBP1). The DSS-induced ER-stress resulted in impaired intracellular trafficking and polarized sorting of sucrase-isomaltase (SI) and dipeptidyl peptidase-4 (DPPIV), which are normally sorted to the apical membrane via association with lipid rafts. The observed impaired sorting was caused by reduced cholesterol levels and subsequent distortion of the lipid rafts. The data presented confirm perturbation of ER homeostasis in DSS-treated Caco-2 cells, accompanied by impairment of membrane and protein trafficking resulting in altered membrane integrity, cellular polarity, and hence disrupted barrier function.

## 1. Introduction

Intestinal homeostasis is maintained through the integrity of the mucosal barrier formed by epithelial cells and their secretions. Several studies have implicated decreased intestinal barrier function in inflammatory bowel disease (IBD) [1,2]. Mutations in various genes, such as nucleotide-binding oligomerization domain-containing protein 2 (NOD2), which plays a role in activating NFҡB, have also been linked to barrier defects in IBD [3,4]. Biological membranes possess defined functional microdomains, or lipid rafts (LR), in which signaling proteins reside. These membranes are liquid-ordered lipid–protein assemblies enriched in cholesterol and sphingolipids that float within the liquid-disordered bilayer of cellular membranes [5]. LRs are resistant to solubilization with non-ionic detergents at low temperatures and can therefore be separated from other detergent-soluble membrane structures by virtue of their low buoyant density in sucrose-density gradients [6]. It has been shown that in an inflamed milieu, cells exhibit lipid raft alterations [7]. Nevertheless, relatively little is known about the potential regulatory influence of LRs and non-lipid raft domains of the cell membrane on epithelial adhesion and barrier function under inflammatory conditions.

Intestinal epithelial cells, which have been shown to be functionally impaired in IBD, can trigger an inflammatory response [8]. In a mouse model, knocking down XBP1, a central regulator of endoplasmic reticulum (ER) stress, results in impaired epithelium function [9]. These mice spontaneously develop small bowel inflammation that markedly resembles some features observed in human IBD, including infiltration of immune cells and ulcerations [9]. In many cases of human IBD, increased ER stress has been observed in inflamed areas of the intestine [10]. However, the signaling pathways that link ER stress to inflammation and intestinal barrier dysfunction are not completely understood.

The ER is the site of protein translocation and folding as well as lipid biosynthesis, which is associated with ER homeostasis. Upon disturbances of ER homeostasis due to altered glycosylation, Ca^2+^ depletion, increased mRNA translation and inflammatory stimuli, unfolded and misfolded proteins accumulate in the ER lumen, leading to ER stress [11]. 

One hypothesis that links ER stress to IBD suggests impaired trafficking of junctional proteins. In dextran sulfate sodium (DSS)-treated mice, a well-accepted model for IBD, epithelial barrier disruption with neutrophil and macrophage infiltration was observed in addition to abnormal cytokine production [12,13]. Similarly, in the study presented here, DSS was used to induce barrier disruption in Caco-2 cells to facilitate investigations into the resulting up- and downstream events attributed to IBD. Caco-2 cells have been reported to be an in vitro model for the intestinal barrier due to their ability to differentiate into a monolayer that exhibits properties typical for absorptive enterocytes [14,15]. We show a correlation between DSS-induced disruption of ER homeostasis, cell membrane composition, and cellular barrier. In this experimental cell model, DSS upregulates ER stress markers and alters lipid raft composition and barrier function. These investigations provide important clues regarding mechanisms by which ER stress compromises lipid raft structure and function in disease states such as IBD. 

## 2. Results

### 2.1. DSS Affects Membrane Integrity

**Figure 1:**
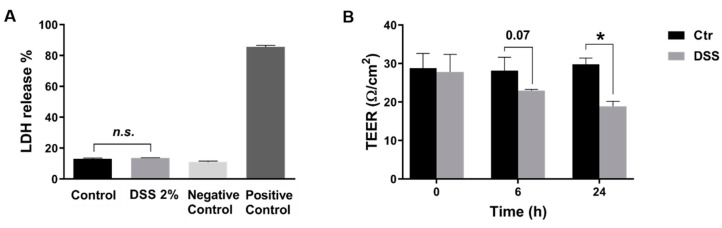
Dextran sulfate sodium (DSS) treatment disrupts intestinal epithelial barrier without affecting cell viability. (**A**) DSS does not affect the viability or the morphological characteristics of Caco-2 at a concentration of 2% (*w/v*), for 24 h, as assessed by the lactate dehydrogenase (LDH) assay. Negative control contains only media (without cells); positive control cells were lysed prior to adding the LDH reaction mix (**B**) The integrity of Caco-2 cells monolayer treated with DSS was measured by trans-epithelial electrical resistance (TEER). Students *t*-test, * *p* < 0.05, SEM, *n* = 3.

In the present study, we evaluated DSS treatment in inducing ER stress in Caco-2 cells. We first tested the potential cytotoxicity of DSS (500 kDa) on these cells. The results obtained revealed that the survival of cells exposed to DSS at 2% (*w/v*) was comparable to that of controls (Figure 1A). Further experiments reported here were performed using this concentration of DSS. 

The next question addressed was how DSS affects the polarity and the epithelial integrity of Caco-2 cells. For this purpose, five days post-confluent Caco-2 cells were treated with DSS, and the trans-epithelial electrical resistance (TEER) was evaluated over a period of 24 h. Figure 1B depicts the results of these experiments and demonstrates a significant decrease in the TEER values of DSS-treated Caco-2 cells by approximately 37% after 24 h. Additionally, the osmotic pressure of the DSS solution used (2%) was 320 mOsm/kg, which is not significantly higher than the osmotic pressure of the cell medium alone (325–500 mOsm/kg; data are not shown). 

**Figure 2:**
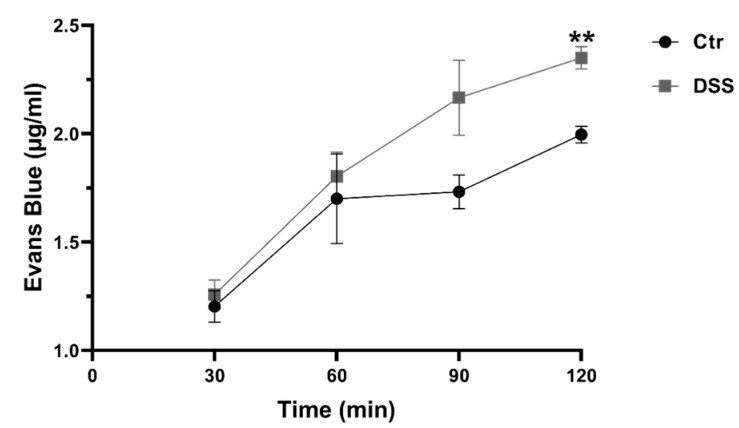
Dextran sulfate sodium (DSS) treatment alters the permeability of the intestinal epithelial barrier. The permeability of the epithelial barrier of Caco-2 cells was evaluated by Evans Blue (EB) permeability assay. DSS affected the cellular integrity negatively and caused a decrease in monolayer integrity as compared to the control (Ctr) non-treated cells. Student’s *t*-test, ** *p* < 0.01, SEM, n = 3.

To evaluate the influence of DSS on epithelial integrity, the Evans Blue (EB) permeability assay was used to test its effect on the permeability of Caco-2 cells monolayer. The level of cell permeability or leakage was correlated to the concentration of EB measured at the bottom of the well. As shown in Figure 2, after 2 h incubation with EB, the concentration of EB significantly and rapidly increased under DSS treatment. This result indicates an increased permeability and an alteration of the epithelial barrier. These effects were not due to DSS cytotoxicity as demonstrated above (Figure 1A).

### 2.2. DSS Induces ER Stress

**Figure 3:**
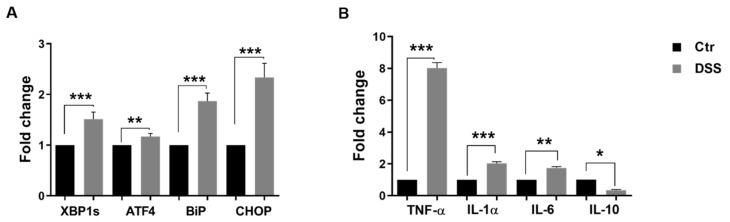
Dextran sulfate sodium (DSS) induces the expression of ER stress markers in Caco-2 cells and changes the balance of pro-inflammatory/anti-inflammatory cytokines. (**A**) Examination of ER stress markers by semi-quantitative RT-PCR. The ER markers X-box binding protein 1s (XBP1s), activation transcription factor 4 (ATF-4), immunoglobulin-binding protein (BiP), and C/EBP homologous protein (CHOP) were significantly elevated in Caco-2 cells incubated with DSS. (**B**) The expression of the pro- and anti-inflammatory cytokines, tumor necrosis factor-α (TNF-α), interleukin α (IL1α), interleukin 6 (IL6), and interleukin 10 (IL10). DSS treatment caused an elevation in the expression of the pro-inflammatory cytokines TNF-α, IL1α, IL6, whereas that of the anti-inflammatory cytokine IL10 was reduced as compared to control (Ctr) non-treated cells. Student’s *t*-test, * *p* < 0.05, ** *p* < 0.01, *** *p* < 0.001, SEM, *n* = 3.

We analyzed the transcription levels of several ER stress markers in five days post-confluent Caco-2 cells by semi-quantitative RT-PCR analysis after 24 h of treatment with DSS. The transcription levels corresponding to the XBP1s, ATF4, CHOP, and BiP proteins, all of which are involved in the activation of cellular stress responses, were significantly increased (Figure 3A). Cytokines play an important role in the maintenance of barrier function and have been suggested to be responsible for the disruption of the monolayer. Semi-quantitative RT-PCR analysis revealed an increase in the levels of IL-1α, IL-6, and TNFα cytokines upon 24 h of DSS treatment (Figure 3B). By contrast, the transcription level of the anti-inflammatory cytokine IL-10 was significantly decreased. 

### 2.3. The Effect of DSS on Protein Trafficking

**Figure 4:**
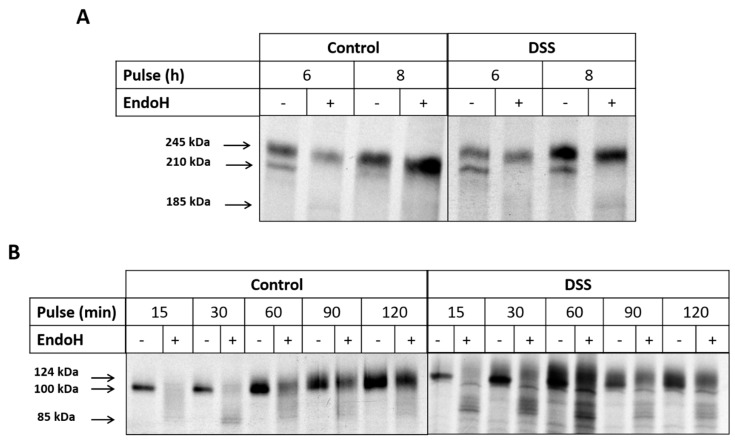
Trafficking of sucrase-isomaltase (SI) and dipeptidyl peptidase-4 (DPPIV) is impaired upon dextran sulfate sodium (DSS) treatment of Caco-2 cells. (**A**) Biosynthetic labeling of SI. Caco-2 cells that were treated or not treated with DSS were labeled for 6 or 8 h with 35S-methionine, and the detergent extracts were immunoprecipitated with anti-SI antibodies. The immunoprecipitates were treated or not treated with Endo H and finally subjected to SDS-PAGE and fluorography. (**B**) Biosynthetic labeling of DPPIV. Caco-2 cells that were treated or not treated with DSS were labeled for 15, 30, 60, 90, and 120 min with 35S-methionine and the cellular extracts immunoprecipitated with anti-DPPIV antibody. Here again, the immunoprecipitated proteins were treated with Endo H and subjected to SDS-PAGE and fluorography.

An impaired function of the ER under stress could influence the trafficking kinetics of proteins from the ER to the Golgi, so we analyzed the trafficking of intestinal sucrase-isomaltase (SI) and dipeptidyl peptidase-4 (DPPIV), which are considered to be typical protein markers that are lipid-rafts associated proteins known to be trafficked with high fidelity across the secretory pathway. The biosynthesis and maturation of these proteins were studied by continuous pulse labeling of DSS-treated or non-treated Caco-2 cells for different time points. The labeling end time points for SI and DPPIV are fixed to a period of time sufficient to reveal the ER-located mannose-rich SI as well as the complex glycosylated mature protein that has been processed in the Golgi apparatus and transported to the apical membrane [16]. The type of glycosylation was assessed by treatment of the immunoprecipitated SI proteins with Endo H. Endo H cleaves mannose-rich N-linked glycans, which characterize ER-located glycoproteins, while mature protein forms that acquire complex glycosylation in the Golgi apparatus are resistant to Endo H. As shown in Figure 4A, after 6 h labeling, two protein bands appear in both treated and non-treated samples, one of which is the 210 kDa mannose-rich SI that is converted to 185 kDa upon Endo H treatment [16] and the upper one (245 kDa) is the Endo H-resistant complex glycosylated mature SI protein. Assessment of the proportions of these biosynthetic forms showed that almost 25% less complex glycosylated SI has been processed in DSS-Caco-2 cells, suggesting that a delay in the processing of the mannose-rich protein form has occurred in the ER. After 8 h labeling and under normal conditions, SI was completely processed and converted into the Endo-H resistant mature SI, while its counterpart in DSS-treated cells did not fully mature and an appreciable proportion (27%) of de novo synthesized SI persisted as a mannose-rich Endo H-sensitive glycoproteins. These data showed that the ER exit of SI is partially impaired in DSS-treated Caco-2 cells. Next, we asked whether these effects are not restricted to SI but may also affect other glycoproteins that are trafficked along the constitutive secretory pathway. For this purpose, we investigated the biosynthesis and processing of DPPIV, a serine exopeptidase that is expressed in intestinal cells and many other cell types. The biosynthetic labeling shows that the conversion of DPPIV to a complex glycosylated mature form is altered by DSS treatment (Figure 4B). DPPIV is transported between the ER and the Golgi at a faster rate than SI [17]. Therefore, we chose short labeling time points to detect early forms of DPPIV in the ER and chase these forms to maturation through the Golgi apparatus. As shown in Figure 4B, 15 and 30 min into the chase, DPPIV was still found in the ER as a mannose-rich Endo H-sensitive protein. After 60 min of chase, minor amounts of the 100 kDa form was detected, whereas later on (90 min and 120 min), DPPIV was converted completely to a complex glycosylated Endo H-resistant glycoprotein. By contrast, in DSS-treated cells, Endo H produced multiple protein bands of varying apparent molecular weights at around 85 kDa throughout the chase time points in addition to the 124 kDa mature Endo H-resistant band. This pattern persisted at the 90 and 120 min chase time points, indicating that an appreciable proportion of DPPIV in the DSS-treated cells maintained an Endo H-sensitivity concomitant with reduced trafficking efficiency between the ER and Golgi apparatus.

**Figure 5:**
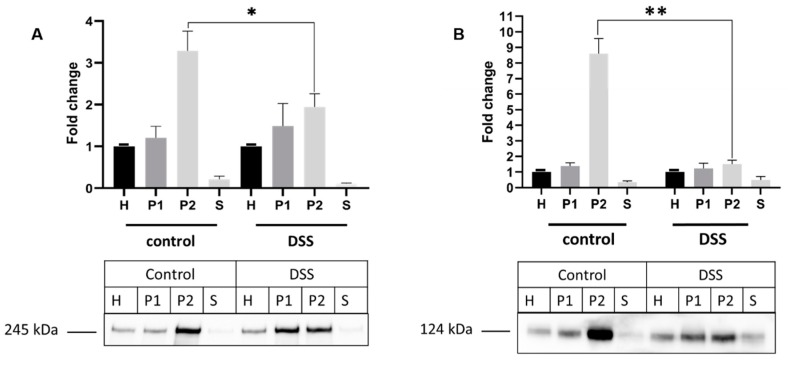
The sorting of sucrase-isomaltase (SI) and dipeptidyl peptidase-4 (DPPIV) is impaired in dextran sulfate sodium (DSS) treated Caco-2 cells. (**A**) Trafficking of SI to brush border membrane (BBM). (B) Trafficking of DPPIV to BBM. Caco-2 cells were treated or not treated with DSS. The cellular homogenates (H) were separated into intracellular and basolateral membranes (P1) or BBM (P2) using CaCl_2_. The fractions were subjected to Western blotting using anti-SI (A) or anti-DPPIV (**B**) antibodies. S represents soluble and vesicular (endosomal) proteins. Upon DSS treatment, a substantial decrease in the expression levels of SI and DPPIV is observed in BBM (P2 fractions). (Student’s *t*-test, * *p* < 0.05, ** *p* < 0.01, SEM, *n* = 3).

We then investigated the effect of DSS on the final trafficking step of SI and DPPIV from the Golgi apparatus to the cell surface. For this purpose, the level of SI expression in brush border membrane (BBM) or apical membrane preparations (P2 fraction) of DSS-treated Caco-2 cells was compared to that in non-treated cells by Western blotting. The results shown in Figure 5A demonstrate an enrichment of approximately 3.5-fold for SI in the P2 fraction in comparison to the overall cellular homogenates (H fraction) in non-treated cells. Upon DSS treatment, a substantial decrease in the level of SI in the P2 fraction was detected, with only a 2-fold increase of its expression in P2 versus H fractions. Concomitant with this decrease is the increase in the SI expression level in the P1 (intracellular and basolateral membranes) fraction of DSS treated cells. 

We further tested the enrichment factor of DPPIV in the BBM upon DSS treatment. Similarly, the enrichment of DPPIV in the apical membrane was decreased to approximately 4-fold versus 7-fold in control untreated cells (Figure 5B).

### 2.4. The Alteration of Membrane Composition Upon DSS Treatment

**Figure 6:**
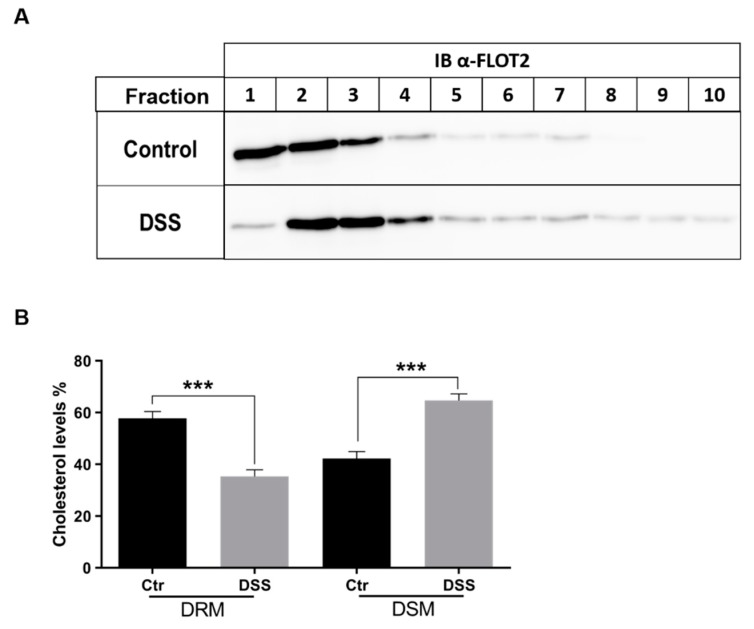
Lipid rafts are altered in dextran sulfate sodium (DSS)-treated cells. (**A**) Control or DSS-treated Caco-2 cells were solubilized with 1% (*w/v*) Triton X-100 and run on sucrose density gradients. Ten fractions were collected and analyzed for distribution of the lipid rafts marker FLOT2 by immunoblotting (IB). FLOT2 is redistributed from fractions 1–3 in control to fractions 2 and 3 (and slightly also 4). The redistribution suggests an altered lipid rafts composition in DSS-treated cells. (**B**) Total lipids were isolated from sucrose gradient fractions obtained from Triton X-100 lysates as described in A). The proportion of cholesterol in the first three fractions (lipid rafts or detergent-resistant membranes/DRM) and in the last three fractions (detergent soluble membranes/DSM) in the WT cells was compared with that of cholesterol from the same fractions in cells treated with DSS. Cholesterol analysis was performed by HPLC (Student’s *t*-test, *** *p* < 0.05, SEM, *n* = 3). A significant reduction (50%) in the cholesterol levels was observed in the DRM of DSS-treated Caco-2 cells.

Lipid rafts (LR) are known to play an important role in the trafficking of SI and DPPIV. We therefore investigated the status of LR under ER stress conditions upon DSS treatment. The lipid rafts were isolated from Triton X-100 cellular extracts of DSS-treated or non-treated Caco-2 cells that were analyzed on sucrose density gradients. LR were recovered in the floating fractions of the gradient as assessed by the distribution of Flot2, a lipid raft scaffold protein marker [18,19] (Figure 6A). Flot2 appeared primarily in the upper three fractions of the gradient and, to a lesser extent, in fractions 4-7. In DSS-treated cells, a clear redistribution of Flot2 to fractions 2 and 3, and slightly also to fraction 4, was observed; the remaining gradient fractions also contained faint but definite protein bands. These data suggest that the composition of LR has been altered in the DSS-treated cells. Cholesterol is a major component of LR, so we analyzed cholesterol content in LR fractions using HPLC. As shown in Figure 6B, the cholesterol levels were significantly reduced by approximately 50% in the lipid rafts fractions of DSS-treated Caco-2 cells, concomitant with an increase in their level in the detergent soluble or non-lipid rafts fractions. 

**Figure 7:**
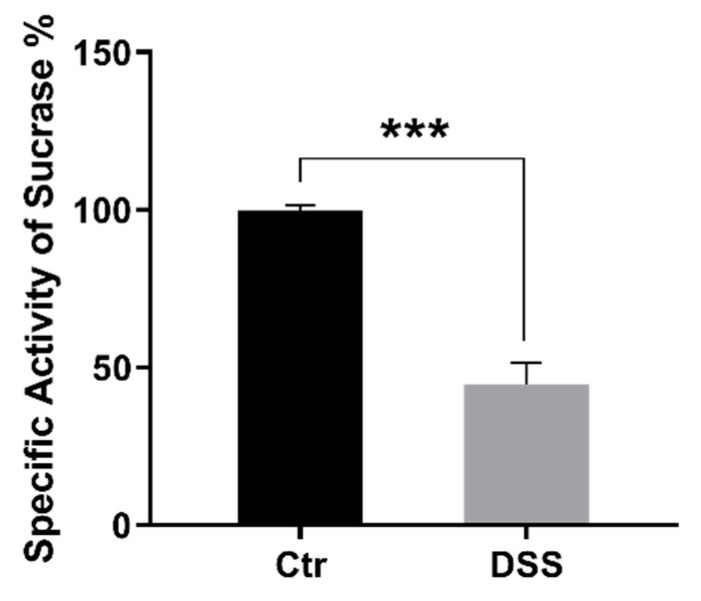
The specific activity of sucrase-isomaltase is reduced in BBM/P2 fractions. Caco-2 cells were treated or not treated with dextran sulfate sodium (DSS). The cellular homogenates were separated into intracellular+ basolateral membranes and brush-border membranes (BBM/P2) using CaCl_2_. The specific activity of sucrase-isomaltase (SI) versus sucrose as a substrate in BBM/P2 fractions was measured. The results revealed a 3-fold approximate significant reduction of SI activity in DSS-treated Caco-2 cells in comparison to the control (Ctr) non-treated cells. Student’s *t*-test, *** *p* < 0.001, SEM, *n* = 3.

Finally, since SI can be found in an active state in both LR and non-lipid rafts, we determined the activity levels of SI versus sucrose as a substrate in P2 fractions of control and DSS-treated cells. Figure 7 shows a 3-fold approximate reduction of SI activity in DSS-treated Caco-2 cells in comparison to the control.

## 3. Discussion

DSS-induced colitis is the most commonly used animal model for IBD even though its precise molecular action is still poorly understood. Recent studies have proposed nuclear targeting to be the mode of action of DSS inducing inflammation in IBD [20]. Caco-2 cells are used as an in vitro model for the intestinal barrier due to their ability to differentiate into a monolayer that exhibits properties typical for absorptive enterocytes [14]. Here, we evaluated the effect of DSS in inducing ER stress and an IBD-like phenotype [12,21,22] in Caco-2 cells. The reported decrease in TEER values, as well as the impaired permeability of the epithelial barrier, are compatible with disruption of tight junctions [23], a situation that also occurs in IBD [24]. Having established the effects of DSS on Caco-2 cells, we next addressed its potential role in inducing ER stress with the ultimate goal of unraveling basic mechanisms that could be altered in this experimental model. The results obtained reveal a significant increase in the transcriptional levels of several ER stress markers involved in activation of cellular stress responses, namely XBP1s, ATF4, CHOP, and BiP proteins. Moreover, cytokines such as interferon γ and tumor necrosis factor α (TNF α,) play an important role in the maintenance of barrier function [25], and several studies have shown that they can be responsible for the disruption of the monolayer [26,27]. Here we could show that Caco-2 cells exposed to DSS treatment exhibit an upregulation in the transcriptional levels of IL-1α, IL-6, and TNFα cytokines, accompanied by a downregulation in the transcription level of the anti-inflammatory cytokine IL-10. This is in line with previously published studies that report the alteration of cytokine production in the intestinal mucosa of pediatric IBD [28]. 

The ER is the most critical organelle in which protein trafficking is regulated in its early stages. Protein folding involving molecular chaperones, acquisition of transport competence, and vesicular budding are key events that occur in the ER and dictate the life cycle of membrane-, secretory-, or lysosomal proteins. One of the ER stress markers that is upregulated in DSS-treated Caco-2 cells is BiP. This molecular chaperone plays a role in the activation of IRE1, PERK, and ATF6 in response to the accumulation of unfolded proteins in the ER [29]. The upregulation of BiP and CHOP, in addition to the transcription factor ATF4 upon DSS-treatment, is compatible with an ER under stress conditions. 

An impaired function of the ER under stress could influence the trafficking kinetics of proteins from the ER to the Golgi. In fact, the biosynthesis and maturation of two intestinal membrane glycoproteins SI and DPPIV, revealed a substantial trafficking delay along the early secretory pathway from the ER to the Golgi apparatus as assessed by the decrease in the proportion of mature, complex glycosylated forms of SI and DPPIV in DSS-treated cells. These two proteins are sorted to the apical membrane with high fidelity via their interaction with cholesterol- and sphingolipids-enriched membrane microdomains or lipid rafts (LR) [30,31]. Interestingly, DSS treatment caused a substantial reduction of SI and DPPIV expression in the brush border membranes concomitant with an increase in the protein levels in the intracellular and basolateral membranes. These results clearly indicate that DSS has impaired the apical sorting event of these proteins, which either were retained intracellularly or diverted their trafficking to the basolateral membrane. In view of the crucial role of LR in the sorting fidelity of SI and DPPIV, an alteration in the composition of the LR due to DSS treatment could negatively impact sorting events and explain the reduced levels of these proteins in the brush border membrane. Our results show a re-distribution of Flot2, a lipid raft scaffold protein marker [18,19], in the LR fraction of the DSS-treated cells concomitants with a significant reduction of the major LR component cholesterol, the biosynthesis of which commences in the ER [32] and is therefore affected by the DSS-induced ER stress. Another piecec of evidence for the distortion of LR is provided by the substantial reduction in the enzymatic activity of SI that is normally higher in LR than in non-LR [33]. Therefore, the trafficking of SI and DPPIV to the apical membrane is partially impaired along the entire secretory pathway, in the ER and in the TGN, where the association with LR and sorting takes place. 

Altogether, DSS treatment has induced ER stress, disrupted monolayer integrity, decreased cell polarity, altered LR, and substantially affected protein trafficking of membrane glycoproteins. Noteworthy, alterations in the trafficking behavior and membrane lipid composition in DSS-treated cells are closely linked to the increased expression of ATF4 and CHOP, which are, in turn, activators of the pro-inflammatory cytokine TNF-α [34,35], which is directly implicated in the pathogenesis of IBD.

## 4. Materials and Methods

### 4.1. Cell Culture

Colon carcinoma Caco-2 cells were obtained from Leibniz Institute DSMZ (Braunschweig, Germany) and cultured in Dulbecco’s Modified Eagle Media (DMEM, Sigma, United Kingdom) supplemented with 10% FCS (Sigma, United Kingdom), 4 mM l-glutamine, 1 mM pyruvic acid, 4.5 mg/mL D-glucose, 100 units/mL potassium penicillin G, and 100 μg/mL streptomycin sulfate in a 5% CO2, 95% air humidified atmosphere. Five days post-confluent Caco-2 cell monolayers were incubated with 2% DSS in DMEM (500 kDa; MP biomedicals GmbH, Germany) for 24 h. 

### 4.2. Measurement of Cell Death

Lactate dehydrogenase (LDH) release assay was used to measure the viability of cells treated with DSS. Cells were cultured in 96 well plates with or without DSS. Control cells were incubated with media only, whereas positive control cells were treated with 1% Triton X-100. Negative control wells contained media without cells. Cell supernatants (100 μL) were mixed with 100 μL CytoTox-ONE™ reagent (Promega, Wisconsin, USA) and incubated for 10 min at 22 °C in a 96-well plate. Fluorescence was recorded with an excitation wavelength of 560 nm and an emission wavelength of 590 nm, according to the manufacturer’s instructions.

### 4.3. Measurement of Trans-Epithelial Electrical Resistance 

Caco-2 cells were cultured on Transwell inserts with 3-μm–pore size filters (Falcon^®^ Cell Culture Inserts, Sterile, Corning^®^). Five days post-confluence, the TEER was measured over a period of 24 h using an EVOM voltmeter with an ENDOHM-12 (World Precision Instruments, Florida USA). Electrical resistance was expressed as Ω × cm2. The TEER was calculated by subtracting the resistance of blank filters from that of filters covered with a monolayer of Caco-2 cells.

### 4.4. Evans Blue Permeability Assay (EBPA)

Evans Blue (EB) solution (Sigma - E2129) was prepared in 1% BSA (bovine serum albumin, cell culture grade; Grand Island Biological Company, New Zealand, Cat# 11020-021) at a concentration of 170 μg/mL then filtered through 0.22-μm filters (Corning). Caco-2 cells were cultured on transparent PET membrane cell culture inserts with 0.4-μm–pore size (Corning^®^) for 5 days post-confluency then treated for 24 h with 2% DSS. After that, cells were washed twice with PBS and EB solution (400 µl) was added on top of the cells while all wells were washed and 1 mL of PBS was added to the bottom. Cells were then incubated at 37 ⁰C, 5% CO2. Every 30 min, 200 µl of PBS from the bottom of each well was collected, followed by the addition of new PBS (200 µl) for 2 h. Optic densities were measured at 630 nm, and concentrations of Evans blue were calculated.

### 4.5. Total RNA Isolation and Reverse Transcription 

Total RNA from five days post-confluent Caco-2 cells was extracted using Trizol reagent as per manufacturer’s instructions (Thermo Fisher Scientific, Waltham, USA). Firstly, cDNA synthesis of total RNA using SuperScript III Reverse Transcriptas (Invitrogen, California USA) was performed according to the manufacturer’s protocols with poly(dT) primer. cDNA fragments encoding the various ER stress markers and cytokines were amplified using a set of sense and antisense primers as listed in Table 1. Semi-quantitative RT-PCR was performed using 1 μL of diluted cDNA template (250 ng) according to published procedures [36] and the PCR products separated by electrophoresis on 2% agarose gels. RPL27 was used as a housekeeping gene.

### 4.6. Biosynthetic Labeling of Sucrase-Isomaltase and Dipeptidyl Peptidase-4 

Five days post-confluent Caco-2 cells were treated with DSS as described previously. Cells were then starved for 2 h in methionine-free media followed by continuous labeling with [^35^S]-methionine. The cells were labeled for different time points, either 6 and 8 h or 15, 30, 60, 90, and 120 min, respectively, for SI or DPPIV analysis. Labeled cells were solubilized for 1 h at 4 °C in lysis buffer (25 mM Tris-HCl, pH 8, 50 mM NaCl, 0.5% Triton X-100, 0.5% sodium deoxycholate) and a mixture of AEBSF, aprotinin, bestatin, E-64, leupeptin and pepstatin A (Sigma Munich, Germany), as described previously [37]. Immunoprecipitation of SI and DPPIV was performed using mAbs HBB2/614/88 [17] and HSI2 [38] and mAb HBB 3/775/42, respectively (the antibodies were provided by Drs. E.E. Sterchi and H.-P. Hauri, formerly at the University of Bern and University of Basel, Switzerland, and Dr. B. Nichols, Baylor College of Medicine, Houston, USA).

### 4.7. Brush Border Membranes Preparation

Calcium chloride precipitation method was used to isolate brush border membranes from Caco-2 cells with or without DSS treatment, as previously described by Naim et al. [16].

### 4.8. Detergent Resistant-Membrane Preparation

Five days post-confluent Caco-2 cells were solubilized with 1% (*w/v*) Triton X-100 in phosphate-buffered saline (pH 7.4) and protease inhibitors cocktail (see above). The cell lysates were then centrifuged at 5000× *g* for 10 min at 4 °C. Cholesterol- and sphingolipids-enriched lipid rafts, which are detergent-resistant membranes (DRMs), were isolated by ultracentrifugation on a discontinuous sucrose gradient as previously described [39]. Ultracentrifugation was performed at 100,000× *g* for 18 h at 4 °C using an SW 40 rotor (Beckman Coulter, Mississauga, ON, Canada). Fractions of 1 mL (typically 10 fractions in total) were collected from the top of the gradient tube. Then, 30 µl of each fraction was used for immunoblotting with antibodies against the LR marker flotillin-2 (FLOT2) (Sigma, Munich, Germany).

### 4.9. Lipid Extraction and Lipid Composition Analysis

Total lipids from the sucrose gradient fractions were extracted by the method of Bligh and Dyer [40] with slight modifications, as described previously [41]. Cholesterol analysis was performed with a Hitachi Chromaster HPLC, as described by Brogden et al. [42,43].

### 4.10. Western Blotting

Equal volumes of the fractions harvested from the sucrose-density gradients were analyzed by SDS-PAGE on 6% or 8% polyacrylamide gels and transferred to PVDF membranes [44]. For Western blotting, the following antibodies were used: mAbs HBB2/614/88 and HBB3/705/60 for SI [17] mAb HBB 3/775/42 for DPPIV [17], and anti-flotillin-2 B-6 antibody (Santa Cruz).

### 4.11. Statistical Analysis

Experiments were carried out in triplicates and repeated at least three independent times. Data are presented as mean ± S.D and compared using Student’s t-test. *P*-values <0.05 were considered to be statistically significant.

## 5. Conclusion

In the present study, we analyzed the consequences of DSS treatment on ER stress in Caco-2 cells, and demonstrated that ER homeostasis is correlated to the maintenance of cellular integrity through regulating cellular proteins and lipids. Furthermore, we demonstrated that alterations in the membrane lipid composition result in impairment of protein trafficking. Even though Caco-2 cells primed with DSS lack the complexity of in vivo models, our model reveals the molecular basis of inflammatory events in the intestinal cells and is suitable to be utilized to understand the cellular processes that involve manipulation of integrity and homeostasis in the intestinal cells. This cellular model is certainly useful in understanding several aspects of protein and membrane trafficking under DSS-induced ER stress and analysis of downstream events that may regulate cellular polarity in intestinal Caco-2 cells.

## Figures and Tables

**Table 1:** List of primers for detecting ER stress markers by real-time PCR.

Primer	Sequence
XBP1 fw XBP1rev	TGGCCGGGTCTGCTGAGTCCG ATCCATGGGGAGATGTTCTGG
ATF4 fw ATF4 rev	GTTCTCCAGCGACAAGGCTA ATCCTGCTTGCTGTTGTTGG
CHOP fw CHOP rev	AGAACCAGGAAACGGAAACAGA TCTCCTTCATGCGCTGCTTT
BiP fw BiP rev	TGTTCAACCAATTATCAGCAAACTC TTCTGCTGTATCCTCTTCACCAGT
TNF-α fw TNF-α rev	CTTCTGCCTGCTGCACTTTG AGCTGCCCCTCAGCTTGAG
IL-1 α fw IL-1 α rev	CTGAAGAAGAGACGGTTGAGTT AGGGCGTCATTCAGGATGAA
IL-6 fw IL-6 rev	GTAGCCGCCCCACACAGA AGCCATCTTTGGAAGGTTCAGG
IL-10 fw IL-10 rev	GCCTTGTCTGAGATGATCCAGT CCACGGCCTTGCTCTTGTT
GAPDH Fw GAPDH rev	CATGGCCTTCCGTGTTCCTA CCTGCTTCACCACCTTCTTGAT
